# D-box-binding protein alleviates vascular calcification in rats with chronic kidney disease by activating microRNA-195-5p and downregulating cyclin D1

**DOI:** 10.17305/bb.2023.10080

**Published:** 2024-08-01

**Authors:** Ye Yao, Kun Zhao, Yan Zhang, Lihui Wang, Wei Shan, Xu Yan

**Affiliations:** 1 Department of Nephrology, The Second Affiliated Hospital of Qiqihar Medical University, Qiqihar, China; 2 Basic Medical College of Qiqihar Medical University, Qiqihar, China; 3 Clinical Laboratory, The Second Affiliated Hospital of Qiqihar Medical University, Qiqihar, China

**Keywords:** D-box-binding protein (DBP), chronic kidney disease (CKD), vascular calcification (VC), microRNA-195-5p, cyclin D1 (CCND1)

## Abstract

Vascular calcification (VC) is a critical complication in chronic kidney disease (CKD), where transcription factors (TFs) and microRNAs (miRs) could potentially play a pivotal role in its pathogenesis and progression. To explore the potential molecular mechanism by which the TF D-box-binding protein (DBP) regulates the miR-195-5p/cyclin D1 (CCND1) axis and its impact on aortic VC in CKD rats, we established a rat model of CKD with VC through a 5/6 nephrectomy procedure. This model was treated with lentivirus overexpressing DBP or CCND1 to analyze their roles in aortic VC. Additionally, an in vitro cell model of VC was induced by high phosphorus. This model underwent transfection with lentivirus overexpressing DBP or miR-195-5p mimic/inhibitor to confirm their regulatory roles in aortic VC in vitro. We assessed the interactions between DBP and miR-195-5p, as well as between miR-195-5p and CCND1. Our results indicated that the expression of DBP and miR-195-5p was reduced, while CCND1 levels were elevated in both the rat and cell models. Overexpression of miR-195-5p inhibited VC in vascular smooth muscle cells (VSMCs). Bioinformatics prediction and dual luciferase assays confirmed that DBP could act as a TF to enhance miR-195-5p expression, with *Ccnd1* identified as a downstream target gene of miR-195-5p. Overexpression of DBP inhibited aortic calcification in CKD rats, whereas overexpression of CCND1 produced the opposite effect. In conclusion, the TF DBP can inhibit CCND1 expression through transcriptional activation of miR-195-5p, thereby preventing VC in rats with CKD.

## Introduction

Chronic kidney disease (CKD), with its high prevalence, remains a major health concern worldwide [1]. A significant occurrence of vascular calcification (VC) and a substantial frequency of cardiovascular events are two crucial complications associated with CKD [2]. VC, correlated with both renal and cardiovascular diseases, leads to higher mortality rates and poorer health outcomes, particularly in individuals with CKD who also exhibit high levels of serum phosphate (Pi) and severe cardiovascular complications [3]. VC is characterized by the deposition of calcium (Ca) and phosphate (P) in the arteries through a cellular-regulated mechanism, involving the transformation of vascular smooth muscle cells (VSMCs) into cells resembling osteoblasts or chondrocytes [4]. Numerous cellular and molecular mechanisms linked to VC in individuals with CKD underscore the importance of investigating pivotal factors involved in the development of VC pathology [5].

Transcription factors (TFs) act as regulators of gene expression and possess the ability to identify distinct DNA sequences under various physiological circumstances [6]. They are extensively involved in the VC process. For instance, the inflammation-related TFs promote calcification in cultured VSMCs [7]. Moreover, D-box-binding protein (DBP), a TF that controls the expression of several cytochrome P450 (*CYP*) genes, is reported to be poorly expressed in CKD [8]. Furthermore, microRNAs (miR) possess the ability to control cell growth, death, stress response, and transformation. They also significantly impact the calcification process and the differentiation of VSMCs into osteoblasts in the thoracic aorta of rats [9]. miR-195-5p was found to be aberrantly expressed in CKD patients with VC [10]. VC could induce changes in miR-195-5p, resulting in cell cycle deregulation [11]. However, the correlation between DBP and miR-195-5p remains under-researched, and the precise function of miR-195-5p in CKD is largely unexplored, necessitating further research efforts. Additionally, miR-195 was found to increase cyclin D1 (CCND1) expression in laryngeal squamous cell carcinoma [12]. CCND1, one of the cell cycle regulators found in the aorta [13], along with the extracellular signal-regulated kinases 1 and 2 (ERK1/2) signaling pathway, participates in the regulation of VSMC proliferation [14]. However, the underlying molecular mechanisms of CCND1 in CKD are still being explored.

The objective of our research was to uncover the underlying mechanisms of DBP and to demonstrate that improving VC could potentially alleviate symptoms associated with CKD. To achieve this, we employed a rat model of CKD, induced by a 5/6 nephrectomy procedure. We further utilized VC cells to explore the expression and regulatory mechanisms of proteins involved in VC, as well as their modulation by DBP.

## Materials and methods

### Bioinformatics analysis

The miRNA dataset GSE130486 and the messenger RNA (mRNA) dataset GSE146638 were retrieved from the Gene Expression Omnibus (GEO) database for our analysis. The miRNA dataset included two normal control rat vascular tissue samples, two rat vascular tissue samples with three days of induced calcification, and two rat vascular tissue samples with six days of induced calcification. The mRNA dataset comprised five normal control rat vascular tissue samples and five CKD rat vascular tissue samples. Differentially expressed genes (DEGs) were identified using the R language “limma” package, applying a threshold set at an absolute log2 fold change (FC) greater than 1 and a *P* value below 0.05.

The prediction of target genes for miRNA was conducted utilizing the TargetScan, miRDB, and microT databases. The TFs binding to miRNA promoter regions were predicted using the PROMO database. Additionally, genes related to VC were identified in the GeneCards database using the keyword “Vascular calcification.”

### Establishment of the CKD VC rat model

In this study, 70 male Sprague–Dawley (SD) rats, sourced from Vital River Laboratory Animal Technology, Beijing, China, were utilized. Their weights ranged between 180 and 220 g, and they were aged between 6 and 8 weeks. The rats were housed in specific pathogen-free (SPF) conditions under laminar flow racks. The temperature was maintained at a constant range of 24 ^∘^C–26 ^∘^C, while the humidity levels were kept between 45%–55%. Throughout the duration of the experiment, the rats had unrestricted access to sterilized food and water.

After a week of adaptive feeding, 60 rats underwent the CKD model construction. The CKD rat model was established through a 5/6 nephrectomy procedure. In addition, 10 rats were used as the control group and did not undergo CDK treatment. Specifically, the rats were anesthetized using 2% isoflurane at a flow rate of 1 L/min O_2_. Throughout the procedure, their heart rates, body temperature, and respiration rates were carefully monitored, maintained at 36 ^∘^C–37 ^∘^C, 40–60 beats per min, and 310–360 respirations per min, respectively. A left abdominal incision was made to expose the kidney, followed by ligation of the upper and middle renal arteries. A week later, a right abdominal incision was made to dissect and remove the right kidney, achieved by ligating the renal pedicle with a 0-gauge wire. Subsequently, the 5/6 nephrectomy was performed. During the sham operation, the renal artery was exposed but not ligated, leaving both kidneys intact. After surgery, the rats were maintained on a normal diet for two weeks. Subsequently, two weeks later, blood was collected from the tail vein to measure serum creatinine (SCr) levels. If the SCr levels in the CKD rats were approximately twice as high as that in the sham-operated rats, the CKD rat model was considered successfully established. The success rate of model construction was 76.7%, resulting in 46 CKD rats.

The rats that were successfully modeled for CKD were fed a high phosphorus and Ca diet (1.8% phosphorus, and 4% Ca; obtained from Guangdong Medical Laboratory Animal Center, Guangdong, China). Additionally, these rats were intramuscularly injected with 1 µg/kg vitamin D3 (three times per week; sourced from Sigma-Aldrich, Burlington, MA, USA), to induce VC. In contrast, the sham-operated rats and the control group rats were maintained on a normal diet (0.9% phosphorus, and 1.2% Ca), and they were intramuscularly injected with saline for a duration of four weeks. Subsequently, each rat in the experimental group was intravenously injected with 1 × 10^9^ plaque-forming units (pfu) of the virus through the tail vein, followed by a second injection two weeks later. A follow-up experiment was conducted six weeks post-injection. Lentivirus vectors overexpressing DBP and/or CCND1 were procured from Shanghai Genechem Co., Ltd. (Shanghai, China).

The rats in our study were allocated into several distinct groups. The first group consisted of ten sham-operated rats that were maintained on a normal diet, serving as a control. For the CKD rats, there were three distinct groups: ten CKD rats that were fed a high phosphorus diet, ten CKD rats with VC that were transfected with a blank control lentivirus, and 20 CKD rats with VC that were transfected with lentivirus overexpressing DBP and/or CCND1. Ten rats were transfected with lentivirus overexpressing DBP and CCND1. The remaining 10 rats were in a completely blank control group.

### Hematoxylin–eosin (H&E) staining

After euthanizing the rats with CO_2_, we collected 1 cm segments of their aortas and immersed them for 12 h in a 4% phosphate-buffered neutral formalin solution (pH 7.4, 0.1 mol/L concentration). Subsequently, the aortic segments were preserved in a 20% sucrose solution. The specimens were dehydrated, paraffin-embedded, and sectioned into 6-µm thick slices. These sections underwent a dewaxing process using sequential xylene-alcohol treatments. For staining, the sections were first stained with hematoxylin (Beyotime Biotechnology, Shanghai, China) for 5 min and then briefly rinsed in tap water. This was followed by exposure to a hydrochloric acid and ethanol solution, and then staining with eosin (Beyotime Biotechnology) for 2 min. Subsequently, the sections were dehydrated using 85% alcohol for 20 s, followed by 95% alcohol for 1 min. This was followed by immersion in anhydrous alcohol I and II for 2 min each. The sections were then subjected to xylene treatment twice, each for a duration of 2 min. Finally, the sections were mounted using neutral resin and observed under an inverted light microscope (DMI3000, Leica, Germany).

### Detection of biochemical indices

For the detection of biochemical indices, blood samples (10 mL each) were collected from the abdominal aorta of rats subjected to different treatments. The concentrations of Ca, P, SCr, serum intact parathyroid hormone (iPTH), and blood urea nitrogen (BUN) in these samples were then measured.

### Von Kossa and Alizarin red S staining

Von Kossa staining was utilized to observe aortic Ca deposition. The tissue sections were first immersed in a 5% silver nitrate solution, followed by 1 h of UV irradiation. Subsequently, the sections were immersed in a 5% sodium thiosulfate solution for 5 min and then restained with a 1% eosin solution for another 5 min. After the removal of impurities and dehydration, the sections were mounted and subsequently examined and captured using an inverted light microscope (Leica DMI3000).

Alizarin red S staining was employed to identify tissue and cellular calcification. Tissue sections and cells, fixed in 4% formaldehyde phosphate buffer for 10 min, were stained with a 2% aqueous solution of Alizarin red S (Sigma-Aldrich) for 5 min. Once mounted, these specimens were observed and photographed using an inverted light microscope (DMI3000, Leica).

### Determination of calcium content

The concentration of Ca ions in the 2-cm segment of aortic tissue nearest to the iliac bifurcation, as well as in the transfected VSMCs, was determined. This was achieved using a Ca^2+^ assay kit (Leagene, Indianapolis, IN, USA), following the manufacturer’s instructions. Briefly, the tissues or cells were homogenized, and the supernatant was isolated via centrifugation. A mixture consisting of 200 µL methyl thymol blue (MTB) solution and 2.5 µL of the sample was then incubated for 10 min at room temperature. Absorbance was measured at 610 nm using an enzyme marker. Additionally, a bicinchoninic acid (BCA) assay was conducted to determine the total protein concentration. The protein concentration of the lysates was used for normalization.

### Determination of alkaline phosphatase (ALP) activity

ALP activity in rat aortic tissues or VSMCs was evaluated using an ALP ELISA kit (NjjcBio, Nanjing, China). The rat aortic tissues were homogenized in a pre-chilled buffer at a 1:10 ratio, and the mixture was ground on ice to create a tissue homogenate. The supernatant was then extracted through centrifugation. Protein content quantification in the homogenate and VSMCs was performed using the BCA assay. For measuring ALP activity, the protein concentration in VSMCs was corrected as per the kit instructions. The assay involved adding the sample and standard to antibody-coated microtiter wells, followed by the addition of horseradish peroxidase (HRP)-labeled antibody. The wells were then incubated at 37 ^∘^C for 4 h. Absorbance values were measured at a wavelength of 450 nm using an enzyme marker (Biotek, Winooski, VT, USA).

### Reverse transcription-quantitative polymerase chain reaction (RT-qPCR)

Relevant RNA was isolated from rat aortic tissue and VSMCs utilizing the Trizol reagent (Invitrogen, Carlsbad, CA, USA). The RNA concentration was measured with a Nanodrop 2000 spectrophotometer (Thermo, St. Louis, MO, USA). For reverse transcription, we utilized the Mir-X miRNA First-Strand Synthesis Kit (638315, Takara, Dalian, China) in combination with the complementary DNA (cDNA) Reverse Transcription Kit (K1622, Reanta, Beijing, China). The resulting cDNA was diluted to a concentration of 50 ng/µL for the subsequent fluorescent RT-qPCR. RT-qPCR was performed utilizing an ABI 7500 qPCR instrument (Thermo) under the following conditions: a 95 ^∘^C pre-denaturation step for 10 min, followed by 45 PCR cycles. Primers were designed using the National Center for Biotechnology Information (NCBI) database. β-actin was used as the internal reference for mRNA, and U6 was used as the internal reference for miRNA. The 2^−ΔΔCt^ method was employed to assess the expression of target genes. Details of all primer sequences are provided in Table S1. The primers were supplied by GenePharma (Shanghai, China).

### Immunohistochemistry (IHC)

The rat aortic tissue sections were first deparaffinized and rehydrated. They were then immersed in beakers containing potassium citrate solution, followed by microwave antigen thermal restoration at 90 ^∘^C for 10 min. To deactivate endogenous peroxidase activity, the sections were gradually treated with 3% H_2_O_2_ at room temperature for 10 min. After washing with phosphate-buffered saline (PBS), the sections underwent a 20-min blocking step at room temperature using 5% goat serum (Solarbio, Beijing, China). Post-blocking, the sections were incubated with diluted primary antibodies of DBP (1:100; AI10247, Abcepta, China) and CCND1 (1:100, ab16663, Abcam, Cambridge, UK), overnight at 4 ^∘^C. The sections were then exposed to an appropriate quantity of goat anti-rabbit secondary antibody (ZSGB-BIO, China) for 1 h at 37 ^∘^C. Subsequently, they were stained using 3,3′-diaminobenzidine (DAB; ZSGB-BIO) for 3–5 min. For image analysis, the versatile system from Media Cybernetics, Rockville, MD, USA, was employed to manage and analyze cell images in true color. Each specimen was divided into four sections, and three random fields of view were captured for each section. The quantification of cells exhibiting positive staining was conducted using light microscopy. The mean values of these quantifications were used to represent the expression levels of DBP and CCND1, respectively.

### In situ hybridization

Paraffin sections of rat aortic tissues were subjected to deparaffinization and rehydration. The detection of miR-195-5p was carried out using an in situ hybridization kit (Boster, Wuhan, China), with probes designed and synthesized by RiboBio (Guangzhou, China). The sections were treated with freshly diluted 3% citric acid pepsin for 30 min at 37 ^∘^C, followed by fixation in 1% paraformaldehyde containing diethyl pyrocarbonate (DEPC) (pH 7.2–7.6) for 10 min at room temperature. For the pre-hybridization step, 20 mL of 20% glycerol was added to the bottom of the hybridization cassette. Each section was then incubated with 20 µL of pre-hybridization solution for 6 h at 38 ^∘^C–41 ^∘^C, with excess liquid aspirated afterward. Hybridization was performed overnight at 38 ^∘^C–41 ^∘^C after covering each section with protective film and adding 20 µL of hybridization solution. The following day, the sections were incubated with blocking solution for 30 min at 37 ^∘^C, and then incubated with biotinylated murine anti-digoxin for 60 min at the same temperature. This was followed by a 20-min dropwise incubation with streptavidin–biotin complex (SABC) (1:300) at 37 ^∘^C, and subsequently with biotinylated peroxidase for the same duration and temperature. After development with DAB and counterstaining with hematoxylin, the sections were dehydrated with alcohol, cleared with xylene, and finally mounted. Specimen analysis was conducted using a versatile cell image analysis management system (Media Cybernetics, USA) capable of true color functionality. Four sections were selected from each specimen, and three random fields of view were captured for each section. Positively stained cells were counted using light microscopy, and the mean value of these counts was recorded.

### Isolation and culture of primary cells from rat VSMCs

Four-week-old male SD rats, weighing between 80–100 g, were obtained from the Shanghai Experimental Animal Center of the Chinese Academy of Sciences (Shanghai, China). The aorta was surgically removed from the rats under aseptic conditions within an ultra-clean bench. The aorta was then placed in a sterile culture dish and repeatedly rinsed with D-Hank’s solution. The extravascular connective tissues were carefully removed, while the aorta was cut open, followed by the removal of the inner and outer membranes. The vascular membrane was cut into several tissue pieces, approximately 1 mm^3^ in size. These tissue fragments were placed in the culture dish, spaced at intervals of 0.2–0.5 cm, and incubated in a 5% CO_2_ incubator at 37 ^∘^C for 1 h. Following the incubation, the adherent cells in the culture dish were supplemented with Dulbecco’s Modified Eagle Medium (DMEM) containing 20% FBS (Gibco, USA). The culture medium was first changed after five days and then refreshed every three days thereafter. The cells were validated for VSMC identity (positive rate > 90%) using immunofluorescence with an anti-alpha smooth muscle actin antibody (ab7817, Abcam). Cells between passages 3–8 were utilized for subsequent experiments.

### High phosphorus-induced VC cell model

Rat VSMCs at passages 3–8, in the logarithmic growth phase, were seeded in 6-well culture plates at a density of 1 × 10^5^ cells/well after trypsin digestion. Subsequently, these cells were cultured for 24 h until they reached approximately 90% confluence. The VC-modeled cells were cultured in DMEM supplemented with 2.5-mM inorganic P (Sigma-Aldrich) and maintained in a 5% CO_2_ incubator at 37 ^∘^C for 8 days, with the medium being refreshed every three days. Meanwhile, the normal control cells were incubated in DMEM containing 1.4-mM inorganic P.

### Cell transfection

At 24 h prior to introducing the cells, rat VSMCs were seeded in 6-well cell culture plates, each containing 2 mL of DMEM complete medium. These plates were incubated at a constant temperature of 37 ^∘^C with a 5% CO_2_ atmosphere. Cell transfection was carried out when the cellular confluence reached 80%. The plasmids of miR-195-5p mimic, mimic-negative control (NC), inhibitor-NC, and miR-195-5p inhibitor were obtained from RiboBio. Additionally, the lentivirus DBP blank vector and overexpression vector were obtained from GeneChem. The overexpression lentiviral vector used was GV287 (Genechem), featuring a 10.4-kb sequence incorporating the ampicillin resistance gene, green fluorescent protein marker, and FLAG tag protein. The GV248 (GeneChem) was utilized as the interfering lentiviral vector plasmid. The lentivirus was introduced to the cell culture plates at a multiplicity of infection (MOI) of 5, in accordance with the manufacturer’s instructions for viral infection. The lentivirus was incubated with the cells for 48 h for use in subsequent experiments.

For control conditions, VSMCs were cultured under normal circumstances. In contrast, VSMCs cultured under high *P* conditions were designated as VC cells. Concurrently, VSMCs were transfected with overexpression blank vector lentivirus, DBP overexpression lentivirus, plasmids of inhibitor-NC, miR-195-5p inhibitor, mimic-NC, and/or miR-195-5p mimic.

### Chromatin immunoprecipitation (ChIP)

ChIP was carried out using the EZ-Magna ChIP TMA kit (Millipore, Billerica, MA, USA). VSMCs in their logarithmic growth phase were treated with 1% formaldehyde for 10 min to cross-link the chromatin. This was followed by quenching with 125-mM glycine for 5 min at room temperature. The cells were subsequently gathered through centrifugation at 2000 *g* for 5 min. A cell lysate solution was prepared, bringing the cell concentration to 2 × 10^6^ cells/200 µL. The cells underwent protease inhibitor treatment and subsequent centrifugation at 5000 *g* for 5 min. The cells were resuspended in a nuclear separation buffer and lysed in an ice-cold water bath for 10 min. Subsequently, sonication was performed until the desired chromatin fragments ranging from 200–1000 bp were achieved. The cells were subjected to centrifugation at 14,000 *g* for 10 min at 4 ^∘^C in order to obtain the supernatant. Next, 100 µL of this supernatant containing DNA fragments was combined with 900 µL of ChIP dilution buffer and supplemented with 20 µL of a solution consisting of 50 times the concentration of protease inhibitor cocktail (PIC). ProteinA agarose/salmon sperm DNA was added to a total volume of 60 µL, mixed, and incubated at 4 ^∘^C for 1 h. After standing for 10 min, the mixture was centrifuged at 700 *g* for 1 min, and 20 µL of the supernatant was reserved as input. The assayed supernatant was combined with 1 µL of DBP rabbit antibody (Millipore), whereas the NC received 1 µL of rabbit anti-immunoglobulin (IgG; ab172730, Abcam). Each tube received an addition of 60 µL of proteinA agarose/salmon sperm DNA and was gently rotated at 4 ^∘^C for 2 h. Following a 10-min standing and centrifugation at 700 *g* for 1 min, the supernatant was discarded, and the pellet was washed sequentially with 1 mL each of low-salt buffer, high-salt buffer, LiCl solution, and TE buffer (twice each). Two rounds of elution were performed on each tube using 250 µL of ChIP wash buffer. De-crosslinking was performed with 20 µL of 5M NaCl followed by DNA recovery. The enriched chromatin fragments were detected using fluorescence qPCR with specific primers for the miR-195-5p promoter: site1 (forward: 5’-GGGGCCTAAAAAGACTGCTT-3’; reversed: 5’-AACCTCCCAGAAGGCAAAAGT-3’), and site4 (forward: 5’-CATGTTTGCCACTCACACCTC-3’; reversed: 5’-CACAGTGCCGTACAAACCAC-3’).

### Dual-luciferase reporter gene assay

The dual-luciferase reporter gene plasmids containing either the wild type (WT) or mutant type (Mut; with a deletion in DBP binding site1) of the miR-195-5p promoter were constructed separately. These reporter plasmids were then co-transfected into VSMCs along with other related plasmids. After 48-h post-transfection, the cells were centrifuged at 12,000 *g* for 1 min. The liquid portion above the sedimented material was then harvested. The luciferase activity was measured utilizing the Dual-Luciferase^®^ Reporter Assay System (E1910, Promega, Madison, WI, USA). For each cell sample, 100 µL of substrate specific to firefly luciferase was added to measure firefly luciferase activity. Subsequently, another 100 µL of substrate specific to renilla luciferase was added to measure renilla luciferase activity. The ratio of the measured values of firefly and renilla luciferase activities was calculated, serving as an indicator of the overall luciferase activity in each sample.

### Western blot assay

The BCA kit (Thermo) was utilized to determine the protein concentration in rat aortic tissues and VSMCs after total protein extraction. For electrophoresis, 30 µg of the protein sample was subjected to sodium dodecyl sulfate-polyacrylamide gel electrophoresis (SDS-PAGE) under a constant voltage of 80 V for 35 min, followed by an increase to 120 V for an additional 45 min. After completion of the electrophoresis, the total proteins were transferred to polyvinylidene fluoride (PVDF) membranes (Amersham, UK). The membranes were then blocked using 5% skim milk powder for 1 h at room temperature. For antibody incubation, the membranes were treated overnight at 4 ^∘^C with diluted primary antibodies of DBP (1:1000, AI10247, Abcepta), CCND1 (1:1000, ab16663, Abcam), runt-related TF 2 (RUNX2) (1:1000, ab236639, Abcam), bone morphogenetic protein 2 (BMP2) (1:1000, ab214821, Abcam), and glyceraldehyde 3-phosphate dehydrogenase (GAPDH) (1:3000, ab8245, Abcam) in a shaker. The membranes were incubated with HRP-labeled secondary antibodies, either goat anti-mouse IgG (ab6789, Abcam) or goat anti-rabbit IgG (ab6721, Abcam), at appropriate dilutions for 1 h at room temperature. Subsequently, the membranes were subjected to scanning and development using an optical luminescence instrument (GE, Boston, MA, USA). The protein band intensities were quantified through grayscale analysis using Image Pro Plus 6.0 software (Media Cybernetics, USA). Additionally, the expression levels of the proteins of interest were normalized to the internal reference protein GAPDH.

### Ethical statement

Ethical approval for this study was obtained from the Animal Ethical Care Committee of Qiqihar Medical University (Approval No. QMU-AECC-2022-74). All procedures in this animal study were conducted in strict compliance with the standards and principles established by the Animal Ethics Committee.

### Statistical analysis

Statistical analyses were conducted using IBM SPSS software, version 21.0 (IBM Co., Armonk, NY, USA). Measurement data are presented as mean ± standard deviation. Initially, the Shapiro–Wilk test was applied to assess the normality of data distribution. For data conforming to a normal distribution, comparisons between the two groups were conducted using the *t*-test. In cases where the data did not follow a normal distribution, the Mann–Whitney *U* test was utilized. For comparisons among multiple groups, a one-way analysis of variance (ANOVA) was employed. A *P* value of less than 0.05 was considered to indicate statistically significant differences.

## Results

### miR-195-5p is poorly expressed in CKD VC models

**Figure 1.**
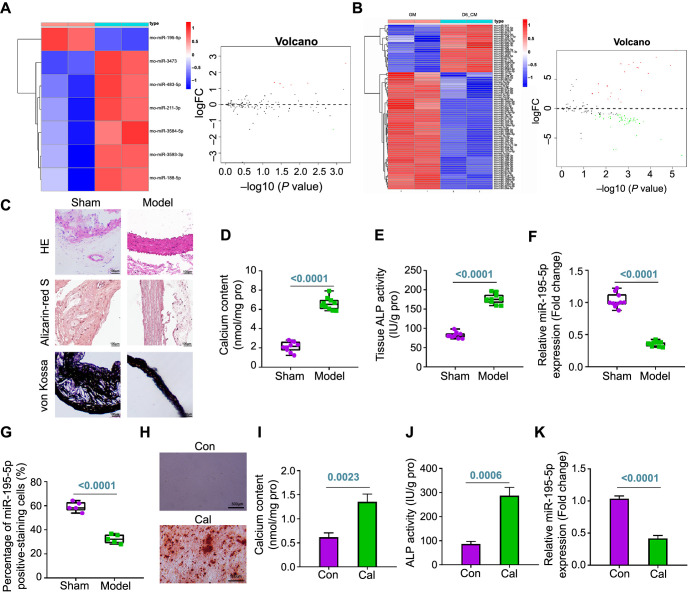
**Expression of miR-195-5p in VC cells and CKD VC rats.** (A) A heat map and volcano plot illustrating differentially expressed miRNAs between control samples and vascular tissue samples from rats with three days of induced calcification in the GSE130486 dataset; (B) A heat map and volcano plot illustrating differentially expressed miRNAs between control samples and vascular tissue samples from rats with six days of induced calcification in the GSE130486 dataset; (C) H&E, Alizarin red S, and Von Kossa staining of aortic tissues (scale bar ═ 100 µm; *n* ═ 5); (D) Calcium quantification in rat aortic tissues (*n* ═ 10); (E) ALP activity detection in rat aortic tissues (*n* ═ 10); (F) RT-qPCR analysis of miR-195-5p expression in rat aortic tissues from each group (*n* ═ 10); (G) In situ hybridization assay for miR-195-5p expression in rat aortic tissues from each group (*n* ═ 5); (H) Alizarin red S staining of calcium salt deposition in VSMCs (scale bar ═ 500 µm); (I) Calcium content quantification results in VSMCs; (J) ALP activity detection in VSMCs; (K) RT-qPCR analysis of miR-195-5p expression in VSMCs. Cell experiments were repeated three times. miR: Micro RNA; VC: Vascular calcification; CKD: Chronic kidney disease; H&E: Hematoxylin–eosin; ALP: Alkaline phosphatase; RT-qPCR: Reverse transcription quantitative polymerase chain reaction; VSMCs: Vascular smooth muscle cells; FC: Fold change; Con: Control; Cal: Calcification.

To investigate the correlation between VC and miRNAs, a differential analysis of the miRNA dataset GSE130486 was conducted. We identified seven differentially expressed miRNAs in VSMCs three days after VC induction, among which only miR-195-5p was notably reduced (Figure 1A). This expression trend persisted in VSMCs six days after the induction of calcification (Figure 1B).

**Figure S1.**
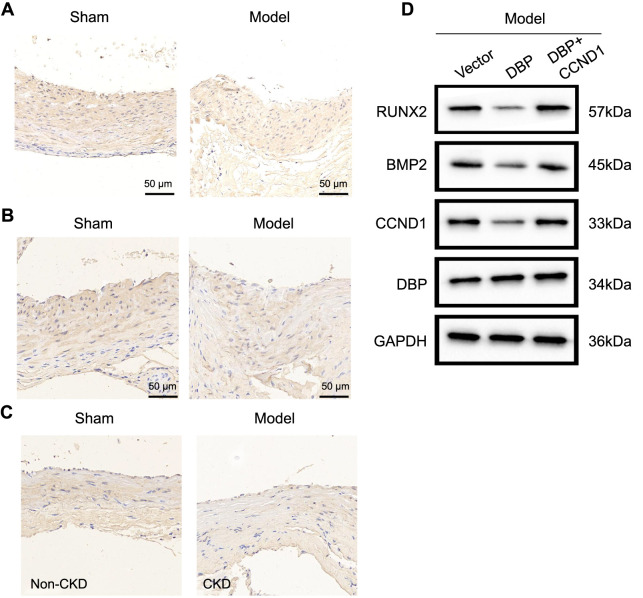
**Staining images and original western blots.** (A) In situ hybridization assay showing miR-195-5p expression in rat aortic tissues in each group (*n* ═ 5); (B) Immunohistochemical detection of DBP protein positivity in rat aortic tissues in each group (scale bar ═ 50 µm; *n* ═ 5); (C) Immunohistochemical detection of CCND1 protein positivity in rat aortic tissues in each group (scale bar ═ 50 µm; *n* ═ 5); (D) Western blot assay evaluating the gene expression of genes related to aortic tissue in each group of rats (*n* ═ 10). miR: MicroRNA; DBP: D-box-binding protein; CCND1: Cyclin D1; CKD: Chronic kidney disease; RUNX2: Runt-related TF 2; BMP2: Bone morphogenetic protein 2; GAPDH: Glyceraldehyde 3-phosphate dehydrogenase.

To validate these findings, a CKD VC rat model was established. In this model, CKD VC rats exhibited significant increases in BUN, Scr, Pi, and iPTH levels, but a decrease in Ca levels (Table S2). Additionally, there was evident disorganization of aortic elastic fibers and pronounced Ca and P deposition in the CKD VC rats (Figure 1C). Increases in Ca content and ALP activity were observed in the aortic vessels of CKD VC rats compared to sham-operated rats (Figure 1D and 1E). Furthermore, miR-195-5p expression was lower in the aortic tissues of CKD VC rats than that in the sham-operated rats, predominantly in the cytoplasm of the smooth muscle layer of the aortic vessels (Figure 1F and 1G; Figure S1A). These findings confirmed the successful establishment of the CKD VC rat model and the significant downregulation of miR-195-5p in this model.

Furthermore, primary rat VSMCs were cultured in a high *P* environment to develop an in vitro VC cell model. These VC cells demonstrated positive Alizarin red S staining, and elevated levels of cellular Ca content and ALP activity (Figure 1H–1J), indicating the successful construction of the in vitro VC cell model. The results obtained from RT-qPCR revealed that miR-195-5p was significantly lower in the VC cells (Figure 1K). Collectively, these findings suggested that miR-195-5p is significantly reduced in both VC cells and CKD VC rat models.

### Overexpression of miR-195-5p inhibits VC in VSMC

**Figure 2.**
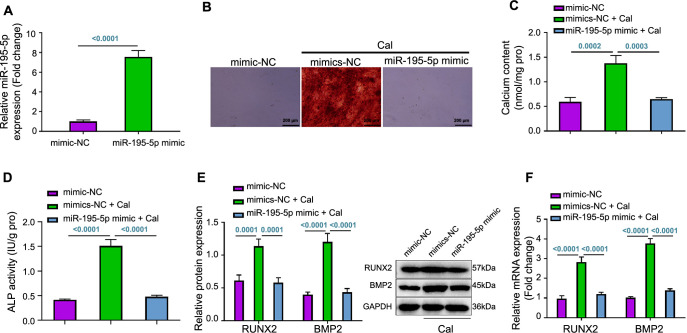
**Effects of miR-195-5p overexpression on VC in VSMCs.** (A) RT-qPCR analysis of miR-195-5p expression in each group of VSMCs; (B) Alizarin red S staining detection of calcium salt deposition in each group of VSMCs (scale bar ═ 200 µm); (C) Measurement results of calcium content in each group of VSMCs; (D) Detection of ALP activity in each group of VSMCs; (E) RT-qPCR and western blot detecting the expression of relevant genes and proteins in each group of VSMCs; (F) RT-qPCR analysis of RUNX2 and BMP2 mRNA expression levels in each group of VSMCs. Cell experiments were repeated three times. miR: MicroRNA; VC: Vascular calcification; VSMCs: Vascular smooth muscle cells; RT-qPCR: Reverse transcription quantitative polymerase chain reaction; ALP: Alkaline phosphatase; RUNX2: Runt-related transcription factor 2; BMP2: Bone morphogenetic protein 2; NC: Negative control; Cal: Calcification; GAPDH: Glyceraldehyde 3-phosphate dehydrogenase; mRNA: Messenger RNA.

To explore the effects of miR-195-5p on VC in VSMCs, this study involved the overexpression of miR-195-5p. RT-qPCR confirmed a significant increase in miR-195-5p levels following the overexpression (Figure 2A). Notably, the extent of Alizarin Red S staining, indicative of Ca deposition, was reduced in calcified VSMCs upon miR-195-5p upregulation. This reduction in Ca deposition was further validated through a cellular Ca content assay (Figure 2B and 2C). Furthermore, the upregulation of miR-195-5p suppressed the increase of ALP activity typically induced by calcification in VSMCs (Figure 2D). Moreover, the upregulation of miR-195-5p also alleviated the increase in the expression of osteogenic differentiation-related factors, specifically RUNX2 and BMP2, in calcified VSMCs (Figure 2E and 2F). Collectively, these findings revealed that the upregulation of miR-195-5p effectively suppressed VC in VSMCs.

### DBP upregulates miR-195-5p expression

**Figure 3.**
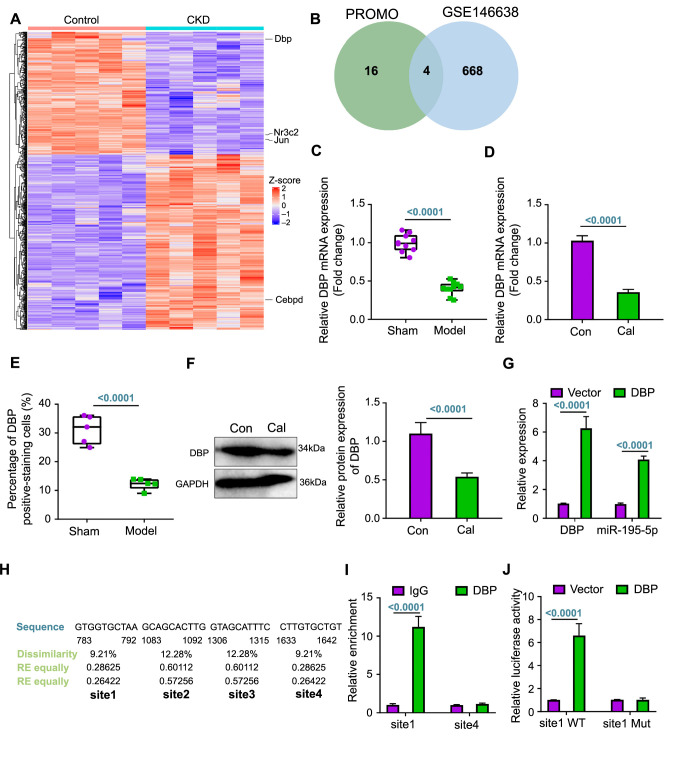
**TF DBP targets and regulates miR-195-5p expression.** (A) A heat map displaying DEGs between control samples (*n* ═ 5) and CKD rat samples (*n* ═ 5) from the GSE146638 dataset; (B) Venn diagram illustrating the intersection of TFs predicted to bind the miR-195-5p promoter region derived from the PROMO database with the DEGs in CKD rats from the GSE146638 dataset; (C) RT-qPCR analysis of DBP expression in rat aortic tissues in each group (*n* ═ 10); (D) RT-qPCR analysis of DBP expression in VSMCs in each group; (E) Immunohistochemical detection of DBP protein positivity in rat aortic tissues in each group (*n* ═ 5); (F) Western blot analysis of DBP expression in VSMCs in each group; (G) RT-qPCR detection of changes in DBP and miR-195-5p expression in each group of VSMCs; (H) Identification of DBP binding sites on the miR-195-5p promoter as predicted by the PROMO database; (I) ChIP assay detecting the DBP enrichment at the miR-195-5p promoter; (J) Dual luciferase reporter gene assay detecting the DBP targeting relationship with miR-195-5p. Cell experiments were repeated three times. TF: Transcription factor; DBP: D-box-binding protein; miR: MicroRNA; DEGs: Differentially expressed genes; CKD: Chronic kidney disease; RT-qPCR: Reverse transcription quantitative polymerase chain reaction; VSMCs: Vascular smooth muscle cells; ChIP: Chromatin immunoprecipitation; IgG: Immunoglobulin G; NC: Negative control; Nr3c2: Nuclear receptor subfamily 3 group C member 2; JUN: Jun proto-oncogene; CEBPD: CCAAT/enhancer binding protein delta; mRNA: Messenger RNA; Con: Control; Cal: Calcification; GAPDH: Glyceraldehyde 3-phosphate dehydrogenase; WT: Wild type; Mut: Mutant type; RE: Random expectation.

To identify TFs that directly regulate miR-195-5p expression, we performed a differential analysis using the GSE146638 dataset (Figure 3A) and intersected with the PROMO database predictions of miR-195-5p regulatory TFs, identifying four candidate TFs (jun proto-oncogene [JUN], nuclear receptor subfamily 3 group C member 2 [Nr3c2], CCAAT/enhancer binding protein delta [CEBPD], and DBP) (Figure 3B). RT-qPCR analysis revealed that DBP was poorly expressed in both the CKD VC rats and VC cells (Figure 3C and 3D), a finding that was consistent with IHC and western blot assay results (Figure 3E and 3F; Figure S1B).

In addition, miR-195-5p levels were distinctly increased after DBP overexpression (Figure 3G). PROMO database analysis revealed the lowest dissimilarity scores for DBP binding at site1 and site4 on the miR-195-5p promoter (Figure 3H). DBP was clearly enriched at the site1 region of the miR-195-5p promoter, but not at site4, implying that site1 is the DBP binding site (Figure 3I). Furthermore, overexpression of DBP promoted the luciferase activity of the miR-195-5p promoter with the site1 WT but not the site1-Mut, suggesting transcriptional activation of miR-195-5p by DBP (Figure 3J). These results suggested that the TF DBP can bind to the miR-195-5p promoter, thereby promoting miR-195-5p transcriptionally.

### miR-195-5p can target and inhibit CCND1 expression

**Figure 4.**
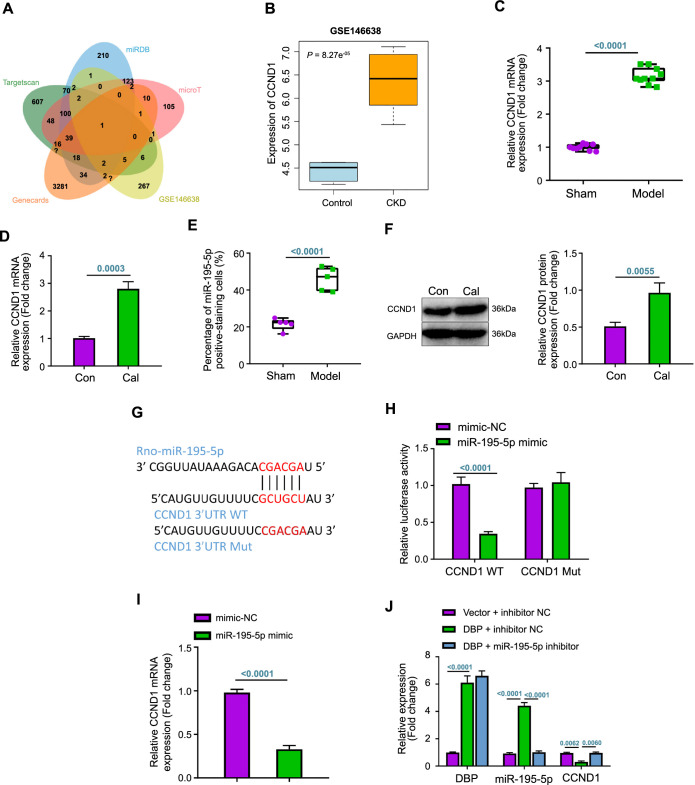
**miR-195-5p targets and regulates CCND1 expression**. (A) Venn diagram illustrating the intersection of miR-195-5p target genes predicted by TargetScan, miRDB, and microT databases with upregulated genes from the GSE146638 dataset and VC-related genes from the GeneCards database; (B) Box plot representing *Ccnd1* expression levels in the GSE146638 dataset, with blue denoting the control group (*n* ═ 5) and yellow representing the CKD group (*n* ═ 5); (C) RT-qPCR analysis of CCND1 expression in rat aortic vessels in each group (*n* ═ 10); (D) RT-qPCR analysis of CCND1 expression in VSMCs in each group; (E) Immunohistochemical detection of CCND1 protein positivity in rat aortic tissues in each group (*n* ═ 5); (F) Western blot analysis of CCND1 expression in VSMCs in each group; (G) TargetScan database predicting CCND1 binding sites to miR-195-5p; (H) Dual luciferase reporter gene assay detecting the targeting relationship between CCND1 and miR-195-5p; (I and J) RT-qPCR analysis detecting the CCND1 expression in each group of VSMCs. Cellular experiments were repeated three times. ^*^Indicates *P* < 0.05 compared to sham, Con, mimics-NC, or vector + inhibitor NC groups; ^#^Indicates *P* < 0.05 compared to DBP + inhibitor NC group. miR: MicroRNA; CCND1: Cyclin D1; VC: Vascular calcification; CKD: Chronic kidney disease; RT-qPCR: Reverse transcription quantitative polymerase chain reaction; VSMCs: Vascular smooth muscle cells; Con: Control; NC: Negative control; DBP: D-box-binding protein; mRNA: Messenger RNA; Cal: Calcification; GAPDH: Glyceraldehyde 3-phosphate dehydrogenase; rno: Rattus norvegicus; WT: Wild type; Mut: Mutant type; 3’UTR: 3’ untranslated region.

To investigate the underlying regulatory pathway of miR-195-5p and identify its downstream processes, we utilized the TargetScan, miRDB, and microT databases to predict the potential target genes. These predictions were then cross-referenced with genes upregulated in CKD rats from the GSE146638 dataset and VC-related genes listed in the GeneCards database. This analysis led to the identification of the candidate gene *Ccnd1* (Figure 4A and 4B).

High expression of CCND1 was observed in the CKD VC rats and VC cells (Figure 4C and 4D), aligning with the results from IHC and western blot assays (Figure 4E and 4F; Figure S1C). Next, we identified the binding site of miR-195-5p on the 3’ untranslated region (3’UTR) of CCND1 using the TargetScan database (Figure 4G). Overexpression of miR-195-5p inhibited the luciferase activity of the *Ccnd1* 3’UTR WT and in the mRNA expression of CCND1, while the luciferase activity of the *Ccnd1* 3’UTR Mut remained unaffected (Figure 4H and 4I). Furthermore, the inhibition of miR-195-5p reversed the inhibitory impact of DBP overexpression on CCND1 expression (Figure 4J). These findings revealed that miR-195-5p can bind to the 3’UTR region of CCND1, thereby inhibiting its expression.

### DBP regulates miR-195-5p/CCND1 axis to prevent VC in CKD rats

**Figure 5.**
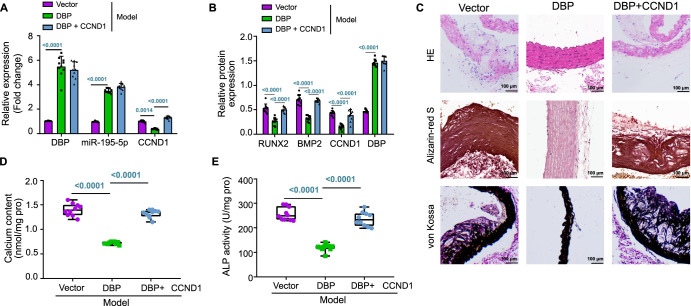
**DBP regulates the effect of miR-195-5p/CCND1 axis on aortic VC in CKD rats.** (A) RT-qPCR analysis evaluating the gene expression of genes related to aortic tissue in each group of rats (*n* ═ 10); (B) Western blot assay evaluating the gene expression of genes related to aortic tissue in each group of rats (*n* ═ 10); (C) H&E, Alizarin red S, and Von Kossa staining of aortic tissue in each group of rats (scale bar ═ 100 µm; *n* ═ 5); (D) Calcium quantification in rat aortic tissues in each group (*n* ═ 10); (E) ALP activity detection in rat aortic vessels in each group (*n* ═ 10). ^*^Indicates *P* < 0.05 compared to vector group; ^#^Indicates *P* < 0.05 compared to DBP group. DBP: D-box-binding protein; miR: MicroRNA; CCND1: Cyclin D1; VC: Vascular calcification; CKD: Chronic kidney disease; RT-qPCR: Reverse transcription quantitative polymerase chain reaction; H&E: Hematoxylin–eosin; ALP: Alkaline phosphatase.

Our experiments led us to hypothesize that DBP might affect the development of VC in CKD rats through the modulation of the miR-195-5p/CCND1 axis. To test this hypothesis, we performed lentiviral transfection to overexpress DBP in CKD VC rats, resulting in the upregulation of DBP and miR-195-5p and a concurrent inhibition of CCND1 expression. CCND1 overexpression reversed the inhibitory effect of DBP upregulation on CCND1. These results were further validated by a western blot analysis (Figure 5A and 5B; Figure S1D).

As shown in Table S3, the levels of P, SCr, BUN, and iPTH were decreased, while Ca levels were increased in rats after DBP overexpression. However, in rats injected with lentivirus overexpressing DBP and CCND1 simultaneously, the levels of P, SCr, BUN, and serum iPTH were elevated while the Ca level was reduced. In addition, DBP overexpression alone alleviated aortic elastic fiber disorder and reduced Ca and P deposition, an effect that was reversed with CCND1 overexpression (Figure 5C).

Further analyses revealed that Ca content, ALP activity, and the expression of osteogenic differentiation-related factors, including RUNX2 and BMP2, were reduced following DBP overexpression. However, these levels were elevated when CCND1 was overexpressed (Figure 5B, 5D, and 5E). These findings indicated that DBP could target and inhibit CCND1 through transcriptional activation of miR-195-5p, thereby preventing VC in CKD rats.

## Discussion

**Figure 6.**
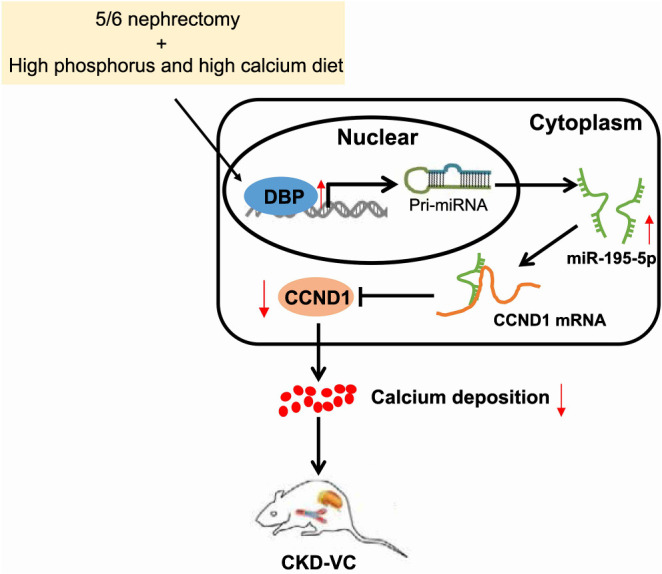
**Illustration of the molecular mechanism by which the TF DBP regulates the miR-195-5p/CCND1 axis affecting VC in the aorta of rats with CKD.** TF: Transcription factor; DBP: D-box-binding protein; miR: MicroRNA; CCND1: Cyclin D1; VC: Vascular calcification; CKD: Chronic kidney disease; pri-miRNA: Primary microRNA; mRNA: Messenger RNA.

VC is a significant factor contributing to the increased risk of cardiovascular disease-related mortality, particularly prevalent in CKD patients with elevated blood P levels [2]. A deeper comprehension of the molecular mechanisms that drive the VC could unveil new avenues for therapeutic interventions. Recent evidence has emphasized the significance of several TFs as crucial regulators in diverse diseases and conditions [15, 16]. Throughout this study, we successfully developed both in vitro VC models and in vivo VC rat models. These models were developed through a 5/6 nephrectomy procedure, followed by a high-phosphorus diet, to simulate VC conditions in CKD. Our research highlights the potential role of the TF DBP as a critical regulator of VC in the context of CKD (Figure 6).

We initially established the CKD VC rat model by performing a 5/6 nephrectomy and feeding the rats a high-phosphorus diet, a method consistent with previous studies in constructing CKD models with VC [17, 18]. Subsequently, we measured various biochemical indices in modeled rats, finding significant increases in the levels of BUN, Scr, Pi, and serum iPTH. SCr has demonstrated its reliability as a biomarker for the breakdown of body protein and/or changes in muscle mass, whereas BUN serves as an indicator for dietary and/or body protein breakdown [19]. Meanwhile, the left ventricular mass index, showing the severity of left ventricular hypertrophy commonly associated with CKD, showed positive correlations with BUN, Scr, and iPTH [20]. Following the successful establishment of the CKD VC rat model, we observed a low expression of miR-195-5p. This miRNA exhibits differential expression in both steatosis and steatohepatitis, making it a commonly observed miRNA [21]. Studies on the association between vascular endothelial function and postpartum whole peripheral blood expression have indicated a role of miR-195-5p in the development of cardiovascular and cerebrovascular diseases [10]. However, the specific functions and mechanisms of miR-195-5p in CKD remain largely unexplored. Our experimental results demonstrated that overexpressed miR-195-5p could inhibit VC in VSMCs. With 122 miRNAs found to be downregulated during calcification progression [22], it is evident that miRNAs play a critical role in the VC process. For instance, upregulated expression of miR-30b has been shown to attenuate VC in vivo [8]. These findings partly align with our results, showing that miR-195-5p is poorly expressed in CKD with VC and that its overexpression can suppress VC.

We further investigated the upstream and downstream mechanism of miR-195-5p, discovering that DBP can increase the miR-195-5p expression. DBP has been previously identified as a TF that falls under the A/B category of heterogeneous nuclear ribonucleoproteins [23]. Consistent with our findings, prior research has also reported reduced DBP expression in CKD mice. DBP is extensively distributed in various tissues, including blood vessels, and plays a role in regulating the expression of multiple genes [24]. However, to our best knowledge, the regulatory relationship between DBP and miR-195-5p has not been extensively explored in existing literature. As we further analyzed, we have found that CCND1 is a target of miR-195-5p, and is negatively correlated with its expression. Coincidentally, miR-195 has been identified as a potential therapeutic target in osteosarcoma treatment due to its ability to inhibit CCND1, thereby exhibiting tumor metastasis suppressor properties [25]. The expression of CCND1 is elevated in the calcification group [26]. Based on these observations, miR-195-5p could be positively regulated by DBP, which in turn negatively mediates the expression of CCND1 in CKD models with VC.

We further analyzed the effect of the identified mechanism on CKD VC rats by performing loss-and-gain of function assays. We found that overexpression of DBP in CKD rats led to a reduction in the expression of RUNX2 and BMP2. Both RUNX2 and BMP2 serve as indicators of the osteoblastic phenotype in VSMCs and are often highly expressed in calcified aortas [27]. Therefore, the reduced levels of RUNX2 and BMP2 suggested a reduction in VC. However, after a simultaneous overexpression of DBP and CCND1, the inhibitory effect of DBP on VC was reversed.

## Conclusion

To summarize, our findings suggested that DBP could play a protective role against VC by regulating the miR-195-5p/CCND1 pathway, as demonstrated in both in vivo and in vitro models (Figure 6). These insights offer promising strategies for the prevention and treatment of VC in CKD. However, the limited exploration of the underlying molecular mechanisms in our study restricts the validity of our results. Additional exploration of CCND1 and VSMCs, along with their individual and combined roles, could lead to a more comprehensive understanding of their mechanisms. Such understanding could provide significant therapeutic implications for managing CKD associated with VC.

## Supplemental data

**Table S1:** Primer sequences for RT-qPCR (rno)

	**Primer sequences**
miR-195-5p	F: 5’-TAGCAGCACAGAAATATTGGC-3’ R: provided by the kit
DBP	F: 5’-GGGACCCACAGTTGCAAAGA-3’ R: 5’-AAATCCTACGAGCACTGCGG-3’
CCND1	F: 5’-TTCTCGTACCACCGGGATCT-3’ R: 5’-CGTCCTGAATCCCCTTGTCC-3’
β-actin	F: 5’-GTGACGTTGACATCCGTAAAGA-3’ R: 5’-GCCGGACTCATCGTACTCC-3’
U6	F: Provided by the kit R: Provided by the kit

RT-qPCR: Reverse transcription quantitative polymerase chain reaction; rno: Rattus norvegicus; miR: MicroRNA; F: Forward; R: Reverse; DBP: D-box-binding protein; CCND1: Cyclin D1.

**Table S2:** Comparative biochemical indices of sham-operated and CKD VC rats

**Groups**	**Ca (mmol/L)**	**P (mmol/L)**	**SCr (µmol/L)**	**BUN (mmol/L)**	**iPTH (pg/mL)**
Sham-operated rats	2.29 ± 0.12	2.09 ± 0.15	28.01 ± 1.15	5.42 ± 0.57	0.71 ± 0.06
CKD VC rats	1.62 ± 0.09^*^	2.43 ± 0.28^*^	52.49 ± 3.50^*^	11.27 ± 0.68^*^	25.94 ± 1.27^*^

*Indicates *P* < 0.05, representing a statistically significant difference compared with the sham-operated rats. CKD: Chronic kidney disease; VC: Vascular calcification; Ca: Calcium; P: Phosphate; SCr: Serum creatinine; BUN: Blood urea nitrogen; iPTH: Intact parathyroid hormone.

**Table S3:** Comparative biochemical indices in rats following vector, DBP, and DBP + CCND1 treatments

**Groups**	**Ca (mmol/L)**	**P (mmol/L)**	**SCr (µmol/L)**	**BUN (mmol/L)**	**iPTH (pg/mL)**
Vector	1.70 ± 0.09	2.48 ± 0.14	54.16 ± 6.86	12.76 ± 0.90	29.65 ± 1.37
DBP	2.46 ± 0.29^*^	2.11 ± 0.23^*^	32.95 ± 1.91^*^	5.98 ± 0.44^*^	5.23 ± 0.90^*^
DBP + CCND1	1.96 ± 0.21^*#^	2.39 ± 0.08^*#^	44.08 ± 3.14^*#^	10.02 ± 1.21^*#^	18.72 ± 2.06^*#^

Vector group represents rats injected with blank empty vector. DBP group represents rats injected with lentivirus overexpressing DBP. DBP + CCND1 group represents rats injected with lentivirus overexpressing DBP and CCND1, simultaneously. ^*^Indicates *P* < 0.05 compared to the vector; ^#^Indicates *P* < 0.05 compared to the DBP. DBP: D-box-binding protein; CCND1: Cyclin D1; Ca: Calcium; P: Phosphate; SCr: Serum creatinine; BUN: Blood urea nitrogen; iPTH: Intact parathyroid hormone.

## Data Availability

The data that support the findings of this study are available from the corresponding author upon reasonable request.

## References

[1] Ene-Iordache B, Perico N, Bikbov B, Carminati S, Remuzzi A, Perna A (2016 May). Chronic kidney disease and cardiovascular risk in six regions of the world (ISN-KDDC): a cross-sectional study. Lancet Glob Health.

[2] Vervloet M, Cozzolino M (2017 Apr). Vascular calcification in chronic kidney disease: different bricks in the wall?. Kidney Int.

[3] Abbasian N (2021 Jul 12). Vascular calcification mechanisms: updates and renewed insight into signaling pathways involved in high phosphate-mediated vascular smooth muscle cell calcification. Biomedicines.

[4] Ning FL, Tao J, Li DD, Tian LL, Wang ML, Reilly S (2022 Mar). Activating BK channels ameliorates vascular smooth muscle calcification through Akt signaling. Acta Pharmacol Sin.

[5] Dube P, DeRiso A, Patel M, Battepati D, Khatib-Shahidi B, Sharma H (2021 Apr 8). Vascular calcification in chronic kidney disease: diversity in the vessel wall. Biomedicines.

[6] Richardson W, Kang GW, Lee HJ, Kwon KM, Kim S, Kim HJ (2021 Aug 24). Chasing the structural diversity of the transcription regulator Mycobacterium tuberculosis HigA2. IUCrJ.

[7] Hénaut L, Sanchez-Nino MD, Aldamiz-Echevarría Castillo G, Sanz AB, Ortiz A (2016). Targeting local vascular and systemic consequences of inflammation on vascular and cardiac valve calcification. Expert Opin Ther Targets.

[8] Hamamura K, Matsunaga N, Ikeda E, Kondo H, Ikeyama H, Tokushige K (2016 Mar 4). Alterations of hepatic metabolism in chronic kidney disease via D-box-binding protein aggravate the renal dysfunction. J Biol Chem.

[9] He L, Xu J, Bai Y, Zhang H, Zhou W, Cheng M (2021 Sep). MicroRNA-103a regulates the calcification of vascular smooth muscle cells by targeting runt-related transcription factor 2 in high phosphorus conditions. Exp Ther Med.

[10] Chao CT, Yeh HY, Tsai YT, Chiang CK, Chen HW (2021 Jul 7). A combined microRNA and target protein-based panel for predicting the probability and severity of uraemic vascular calcification: a translational study. Cardiovasc Res.

[11] Pan W, Yu S, Jia J, Hu J, Jie L, Zhang P (2021 Jun). Deregulation of the cell cycle and related microRNA expression induced by vinyl chloride monomer in the hepatocytes of rats. Toxicol Ind Health.

[12] Duan X, Shen N, Chen J, Wang J, Zhu Q, Zhai Z (2019 Sep 3). Circular RNA MYLK serves as an oncogene to promote cancer progression via microRNA-195/cyclin D1 axis in laryngeal squamous cell carcinoma. Biosci Rep.

[13] Egstrand S, Nordholm A, Morevati M, Mace ML, Hassan A, Naveh-Many T (2020 Dec). A molecular circadian clock operates in the parathyroid gland and is disturbed in chronic kidney disease associated bone and mineral disorder. Kidney Int.

[14] Luo X, Liu J, Zhou H, Chen L (2018 Jul). Apelin/APJ system: a critical regulator of vascular smooth muscle cell. J Cell Physiol.

[15] Zhang Z, Zhang X, Wang C, Zhou P, Xiao J, Zheng H (2021 Oct). Deacetylated Sp1 improves β-glycerophosphate-induced calcification in vascular smooth muscle cells. Exp Ther Med.

[16] Wei X, Su Y, Li Q, Zheng Z, Hou P (2021 Jun). Analysis of crucial genes, pathways and construction of the molecular regulatory networks in vascular smooth muscle cell calcification. Exp Ther Med.

[17] Wang L, Tang R, Zhang Y, Chen S, Guo Y, Wang X (2021 Jun). PTH-induced EndMT via miR-29a-5p/GSAP/Notch1 pathway contributed to valvular calcification in rats with CKD. Cell Prolif.

[18] Wang L, Tang R, Zhang Y, Liu Z, Chen S, Song K (2020 Sep). A rat model with multivalve calcification induced by subtotal nephrectomy and high-phosphorus diet. Kidney Dis (Basel).

[19] Muller TL, Pluske JR, Plush KJ, D’Souza DN, Miller DW, van Barneveld RJ (2022 Sep). Serum creatinine is a poor marker of a predicted change in muscle mass in lactating sows. J Anim Physiol Anim Nutr (Berl).

[20] Wang CJ, Bao XR, Du GW, Wang Y, Chen K, Shen ML (2014 Aug). Effects of insulin resistance on left ventricular hypertrophy in patients with CKD stage 1-3. Int Urol Nephrol.

[21] Vulf M, Shunkina D, Komar A, Bograya M, Zatolokin P, Kirienkova E (2021 Sep 9). Analysis of miRNAs profiles in serum of patients with steatosis and steatohepatitis. Front Cell Dev Biol.

[22] Hromadnikova I, Kotlabova K, Dvorakova L, Krofta L (2020 Jan 9). Evaluation of vascular endothelial function in young and middle-aged women with respect to a history of pregnancy, pregnancy-related complications, classical cardiovascular risk factors, and epigenetics. Int J Mol Sci.

[23] Xu TH, Qiu XB, Sheng ZT, Han YR, Wang J, Tian BY (2019 Aug). Restoration of microRNA-30b expression alleviates vascular calcification through the mTOR signaling pathway and autophagy. J Cell Physiol.

[24] Marsich E, Bandiera A, Tell G, Scaloni A, Manzini G (2001 Jan). A chicken hnRNP of the A/B family recognizes the single-stranded d(CCCTAA)(n) telomeric repeated motif. Eur J Biochem.

[25] Han K, Chen X, Bian N, Ma B, Yang T, Cai C (2015 Apr 20). MicroRNA profiling identifies MiR-195 suppresses osteosarcoma cell metastasis by targeting CCND1. Oncotarget.

[26] Gao M, Chen T, Wu L, Zhao X, Mao H, Xing C (2017 Nov). Effect of pioglitazone on the calcification of rat vascular smooth muscle cells through the downregulation of the Wnt/β-catenin signaling pathway. Mol Med Rep.

[27] Wang Q, Lin P, Feng L, Ren Q, Xie X, Zhang B (2022 Oct). Ameliorative effect of allicin on vascular calcification via inhibiting endoplasmic reticulum stress. Vascular.

